# Professional Secrets

**Published:** 1878-06

**Authors:** 


PROFESSIONAL SECRETS.—The profession of Philadelphia ars endeavoring to effect the passage of the following bill:—"No person duly authorized to practice physic or surgery shall be allowed or compelled to disclose any information which he may have acquired in attending any patient in his professional character, and which information was necessary to enable him to prescribe for such patient as a physician, or to do any act for him as a surgeon."—*Hospital Gazette.*

